# Genome wide analysis reveals genetic divergence between Goldsinny wrasse populations

**DOI:** 10.1186/s12863-020-00921-8

**Published:** 2020-10-09

**Authors:** Eeva Jansson, Francois Besnier, Ketil Malde, Carl André, Geir Dahle, Kevin A. Glover

**Affiliations:** 1 Institute of Marine Research, P. O. Box 1870, Nordnes, 5817 Bergen, Norway; 2 Department of Marine Sciences-Tjärnö, University of Gothenburg, 45296 Strömstad, Sweden; 3 Institute of Biology, University of Bergen, P. O. Box 7803, 5020 Bergen, Norway

**Keywords:** Assignment, *Ctenolabrus rupestris*, Marker validation, Population genomics, Resequencing, SNP

## Abstract

**Background:**

Marine fish populations are often characterized by high levels of gene flow and correspondingly low genetic divergence. This presents a challenge to define management units. Goldsinny wrasse (*Ctenolabrus rupestris*) is a heavily exploited species due to its importance as a cleaner-fish in commercial salmonid aquaculture. However, at the present, the population genetic structure of this species is still largely unresolved. Here, full-genome sequencing was used to produce the first genomic reference for this species, to study population-genomic divergence among four geographically distinct populations, and, to identify informative SNP markers for future studies.

**Results:**

After construction of a de novo assembly, the genome was estimated to be highly polymorphic and of ~600Mbp in size. 33,235 SNPs were thereafter selected to assess genomic diversity and differentiation among four populations collected from Scandinavia, Scotland, and Spain. Global *F*_*ST*_ among these populations was 0.015–0.092. Approximately 4% of the investigated loci were identified as putative global outliers, and ~ 1% within Scandinavia. SNPs showing large divergence (*F*_*ST*_ > 0.15) were picked as candidate diagnostic markers for population assignment. One hundred seventy-three of the most diagnostic SNPs between the two Scandinavian populations were validated by genotyping 47 individuals from each end of the species’ Scandinavian distribution range. Sixty-nine of these SNPs were significantly (*p* < 0.05) differentiated (mean *F*_*ST_173_loci*_ *=* 0.065*, F*_*ST_69_loci*_ *=* 0.140). Using these validated SNPs, individuals were assigned with high probability (≥ 94%) to their populations of origin.

**Conclusions:**

Goldsinny wrasse displays a highly polymorphic genome, and substantial population genomic structure. Diversifying selection likely affects population structuring globally and within Scandinavia. The diagnostic loci identified now provide a promising and cost-efficient tool to investigate goldsinny wrasse populations further.

## Background

Thanks to the rapid development of whole-genome sequencing methods during the last decade [36, 93], genome wide data is now relatively cost-effective to produce and is becoming increasingly commonplace to study evolutionary questions even for non-model organisms [22]. For the conservation and management of wild populations, studies employing high-throughput sequencing technologies may provide better estimates than traditional population genetics tools for key parameters such as effective population size, genetic structure, and connectivity (for reviews, see [2, 85]). Greater resolution from using large numbers of genetic markers and/or by pre-selection of highly divergent markers (i.e., outliers) is especially useful in population genetic studies of marine organisms [33, 83] which are often characterized by very large census sizes and high levels of connectivity leading to generally low levels of population divergence [43]. However, because the efficiency of natural selection is dependent on the (effective) population size [1, 17], adaptive genetic differences are possible – or even likely – in large marine populations inhabiting heterogeneous environments [7, 10, 39]. Therefore, finding biologically meaningful differences among populations, and the possible genetic factors underlying these differences, will help define appropriate management units, and thus sustainably exploit marine species. Better detection and understanding of human-mediated introgression [20], a common problem in many wild fish populations (e.g. [12, 34, 60, 75]), is also of high conservation priority, and now more feasible with the modern sequencing methods at hand.

Goldsinny wrasse, *Ctenolabrus rupestris*, is a small (< 18 cm) inshore marine fish belonging to the *Labridae* family that includes over 500 described species worldwide. It is a common species in the Eastern Atlantic coastal waters from Morocco to Norway, and is also found in the Mediterranean and Black Sea. Traditionally, goldsinny wrasse had no commercial value and thus avoided significant exploitation [26, 41]. However, in response to its increasing demand as a cleaner-fish in the aquaculture industry for delousing cage-reared Atlantic salmon (*Salmo salar*) and rainbow trout (*Oncorhynchus mykiss*), the species is now extensively harvested together with other wrasse species [89]. The demand for cleaner fish in the aquaculture industry has grown almost exponentially after 2007 [88, 89] due to emerged resistance to delousing agents of the salmon louse (*Lepeophtheirus salmonis*) [11, 54]. In Norway alone, ~ 20 million wrasses were caught in 2014–2019 and used in commercial aquaculture. Between 8 to 12 million of these were goldsinny wrasses, which together with corkwing wrasse makes them the numerically most significant of the wild-captured cleaner fish used. In addition to wrasses caught in local waters, Norwegian salmon farms received wrasses caught from the Swedish west coast (about a million fish per year [72];). The high fishing pressure combined with the high breeding-site philopatry of goldsinny wrasse [44], and their slow growth rate (4–5 years for minimum commercial size of 11 cm [88];) indicate that this species is likely to be sensitive to overexploitation. A study by Halvorsen et al. [41] showed that intensive wrasse fisheries could have considerable impact on the target populations: the abundance of goldsinny wrasse was significantly lower on harvested than on control sites (marine protected areas, MPAs) in the Skagerrak region. The authors suggest that such negative fishery effects might be more severe in western Norway, where there are no MPAs and the wrasse fishery is much more intense, and that this reduction in population densities might even lead to cascade effects in the coastal ecosystems. Overexploitation also increases the risk of loss of genetic variation and potentially locally adaptive variants (e.g. [1]). Genetic integrity and adaptability of local populations can also be compromised via manmade gene flow from genetically diverged populations. This concern is of particular importance in fisheries management where large-scale population augmentations through deliberate or inadvertent releases of translocated, captively raised or domesticated individuals occurs [35, 59, 95]. Wild populations of wrasses are not intentionally augmented but receive human-mediated gene flow via large-scale translocations and release or escape of wrasse from fish farms [13, 29].

Given the current practise of using cleaner wrasses in great numbers, and the associated heavy fishing pressure placed on wild populations [41], more knowledge of the population genetic structure of wrasses is required. Thus far, the best-studied of these species is the corkwing wrasse (*Symphodus melops*) which displays clear population subdivisions on different geographic scales [14, 57, 71, 82]. Genetic studies of ballan wrasse (*Labrus bergylta*) have concentrated on investigation of population structure on large geographic scales [4, 21], and on genetic divergence of different morphotypes [3, 80]. A newly published study by Seljestad et al. [84] revealed subdivision on a more local scale as well, dividing the Scandinavian ballan wrasse population into two distinct genetic clusters. An early genetic study of goldsinny wrasse with allozyme markers showed significant differences between the southern and mid-Norway [92]. The only recent study, using a combination of 14 microsatellite and 36 SNP markers [47], revealed clear population divergence (*F*_*ST*_ ~ 0.02–0.05) across the North Sea but only modest differences (*F*_*ST*_ ≤ 0.02) that increased with geographic distance (i.e. isolation-by-distance, IBD) within Scandinavian populations. These patterns are concordant with restricted migration and gene flow as the main factor creating genetic patterns for the species but does not rule out other possible factors such as selection and/or demographic history shaping the population structures seen today (see e.g. [71]). In addition, aquaculture-mediated translocation of wrasses might affect both the donor and the recipient population. The primary area to move wrasses is mid-Norway where there are plenty of fish farms but insufficient local supply of cleaner fish. For corkwing wrasse, which exhibits a clear genetic population subdivision between southern and western Norway (*F*_*ST*_ = 0.107; [14]), it has been shown that translocated fish from southern Scandinavia have escaped and hybridized with local populations in mid-Norway [29]. For goldsinny wrasse, unequivocal determination of escape and/or hybridization with local populations has not yet been possible to determine due to weak level of genetic difference observed between the export and import areas [47].

**Fig. 1:**
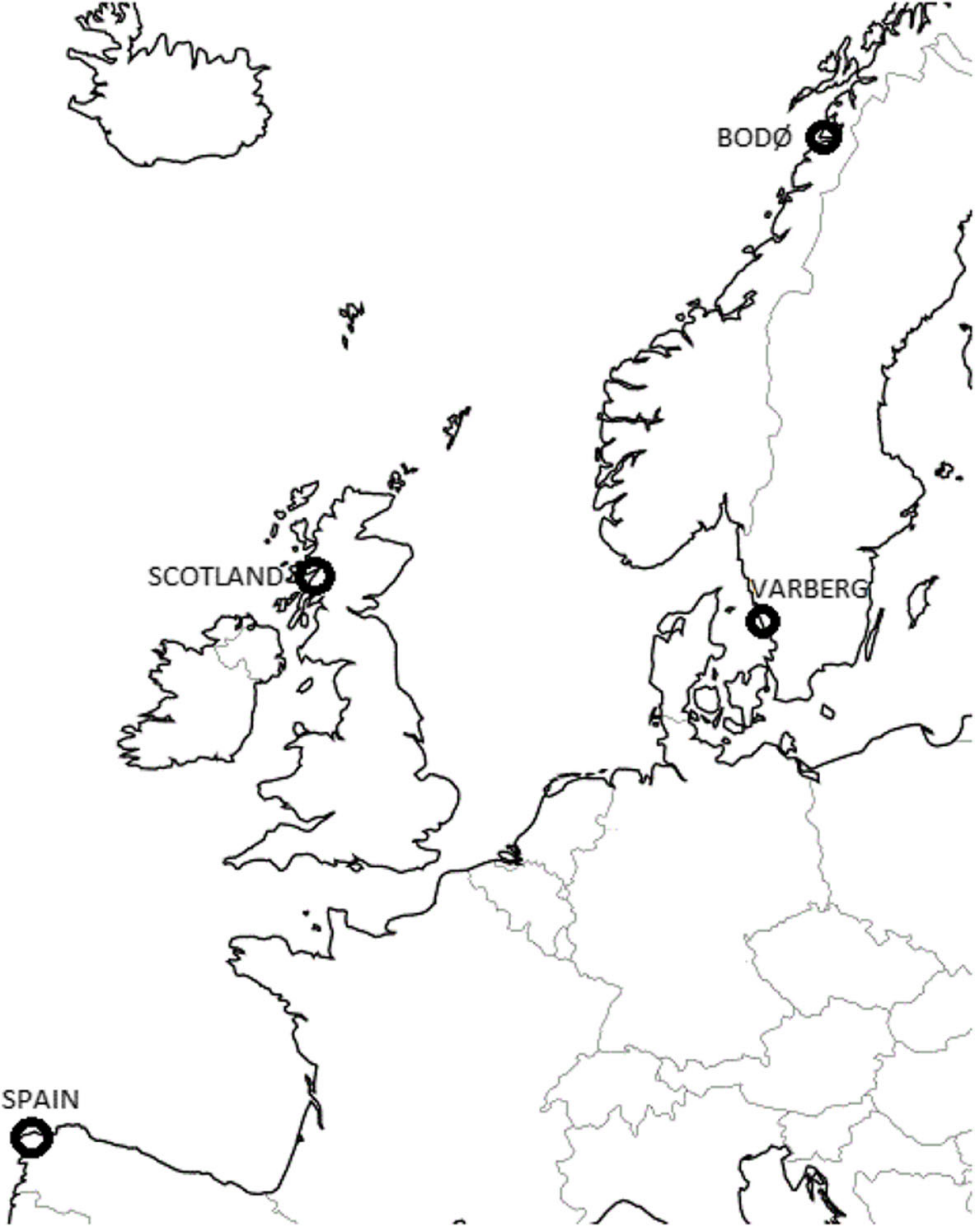
Map of sampling locations. For detailed information, see 5.1

The present study had the following main aims: I) to develop *a* de novo *assembly* for goldsinny wrasse, II) to use a subset of high-quality SNP markers to conduct a first genomic population study for the species using population samples collected along the species entire distribution range (North and South Scandinavia, Scotland and Spain; Fig. 1), III) to compare these results with the results obtained with limited number of markers (in [47]), as well as IV) to identify and validate putatively diagnostic markers between the two Scandinavian populations to be used in future studies.

## Results

### De novo assembled reference

**Fig. 2:**
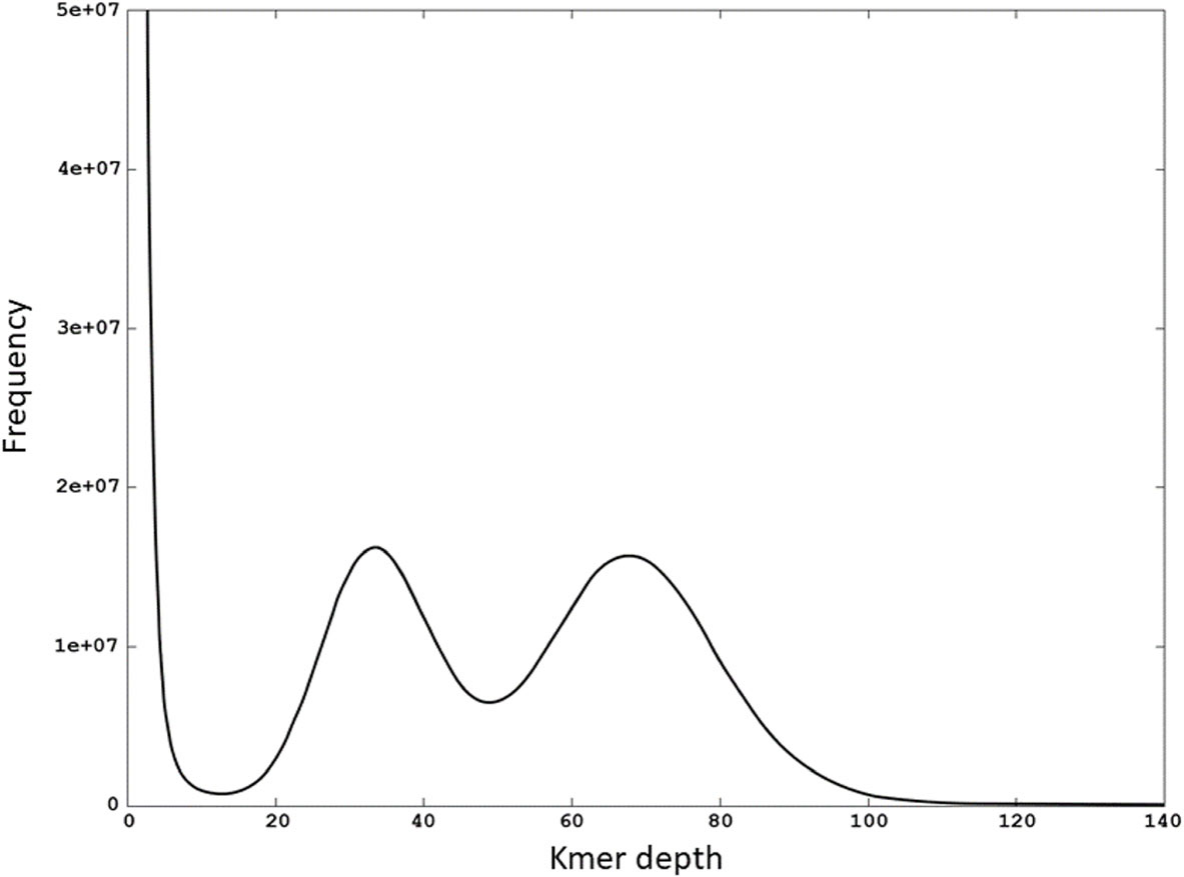
K-mer frequency distribution

The longest continuous scaffolds were obtained using the maximum K-mer size of 127. The best assembly consisted of approximately 3 million contigs (*N* = 2,974,923), with 9.7% reaching 1 kb or longer, and a *N50* of 874 bp. Despite this high level of fragmentation, when reads from all the 60 fish were aligned against the reference, on average, 98.4% (SD ±0.38) were mapped. BUSCO [87] search for the ray-finned fish core genes found 774 complete and 911 fragmented BUSCOs from the reference, comprising 36.8% of the searched genes. Based on the K-mer distribution, estimates for the size of the goldsinny wrasse genome ranged from 580 to 600 Mb. The obtained K-mer profile shows a clear two-peak pattern characteristic to a highly heterozygous genome (Fig. 2). Based on the distribution, the number of heterozygous loci in the goldsinny wrasse reference genome was estimated to be ~ 10 million.

Due to computational constrains that the high fragmentation of the reference causes, SNPs were only called on contigs > 20 kb. There were 1222 such contigs, covering in total ~ 45.4Mbp (~ 7.6% of the genome). Additional information of SNPs (frequency and divergence) in the selected contigs is given in supplementary Table S4.

### Population genomics using 33 k SNPs data set

Genotype correlation was measured between all pairs of loci from the same contig. The average correlation was r^2^ = 0.19 ± 0.09. In comparison, the 100,000 pairs of loci randomly sampled across contigs had an average correlation of r^2^ = 0.12 ± 0.15, indicating a generally higher degree of linkage disequilibrium for the SNPs linked on the same contig compared to SNPs located of different contigs. When excluding the correlation between the nearest n SNPs (with *n* = 1 to *n* = 15), we observed a gradual reduction of LD from r^2^ = 0.18 ± 0.14 (*n* = 5) to r^2^ = 0.16 ± 0.13 (n = 15). The selected loci had an average coverage of 327 ± 100 per site among samples from Bodø, 313 ± 99 from Spain, 329 ± 102 from Scotland and 168 ± 46 from Varberg. Expected heterozygosity across all loci and populations was 0.321, observed heterozygosity 0.319 (Fig. S3), and *F*_*IS*_ 0.006. This overall deficiency of heterozygotes was statistically highly significant (*t* = 43.322, *df* = 33,234, *p* < 2.2e-16), and likely due to population subdivision (see results below). Expected heterozygosity was rather uniform across populations, but realized distribution of variation within populations differed from each other (Table 1). Heterozygote deficiency was observed in the samples from Bodø, Scotland, and Spain, however, the sample from Varberg displayed a clear heterozygote excess.

**Table 1:** Genetic diversity in four goldsinny wrasse populations based on 33 k SNPs and 173 selected loci

	33,235 SNP loci			173 SNP loci		
Population	***N***	***Hs***	***Ho***	***N***	***Hs***	***Ho***
Varberg	15	0.321	0.348	47	0.389	0.313
Bodø	15	0.319	0.307	47	0.385	0.284
Scotland	15	0.325	0.315			
Spain	15	0.319	0.307			
**Mean**		0.321	0.319		0.387	0.299

Populations were clearly differentiated, and the mean *F*_*ST*_ over all four populations based on the 33 k dataset was 0.062, while pair-wise values ranged between 0.015–0.092 (Table 2). Confidence intervals did not include zero in any pair-wise comparison indicating statistically significant differences between all population pairs.

**Table 2:** Pairwise *F*_*ST*_ estimates (below diagonal) and corresponding 95% confidence intervals (above diagonal) based on 1000 bootstraps. Pairwise *F*_*st*_ value and its CI for the Scandinavian Varberg–Bodø pair is also given (in parenthesis) for the putatively diagnostic 173 SNPs genotyped on an extended set of samples

	Varberg	Bodø	Scotland	Spain
**Varberg**		0.023–0.026 (0.050–0.085)	0.078–0.082	0.091–0.095
**Bodø**	0.024 (0.065)		0.071–0.075	0.082–0.085
**Scotland**	0.080	0.073		0.014–0.017
**Spain**	0.092	0.084	0.015	

**Fig. 3:**
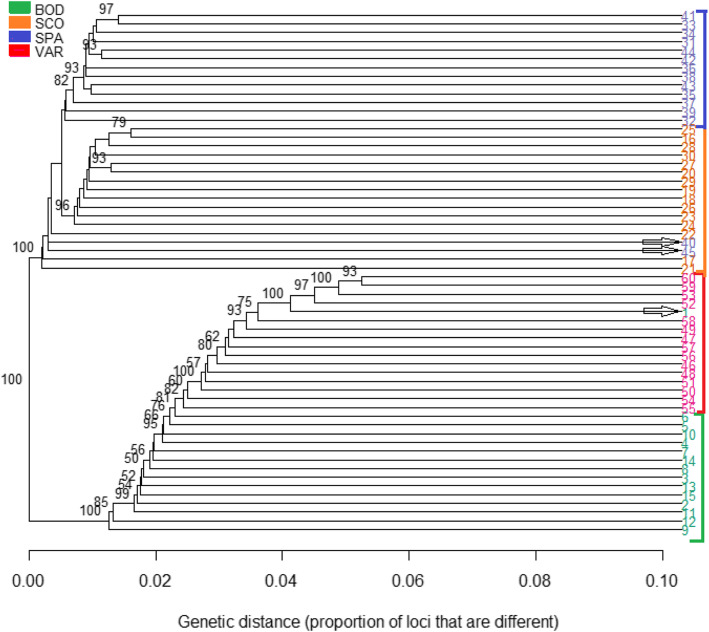
Genetic distance tree between samples based on 33,235 SNPs. Branch nodes supported by ≥50% of bootstrap replicates are shown. Samples clustering with other populations than own are marked with arrows

The pairwise genetic distances between samples showed clear, highly supported (100% of bootstraps) divergence into East Atlantic (Spain and Scotland) and Scandinavian clades (Fig. 3). The majority of individuals in each population clustered together with few exceptions: two individuals (40, 45) from Spain had the closest resemblance to basal Scottish samples, and one sample (1) from northern Norway, Bodø clustered together with Varberg samples from southern Scandinavia.

Inspecting clustering patterns within the main clades, some interesting patterns appeared. First, East Atlantic populations were clearly divided into two populations (Spain and Scotland), but many individuals within these populations were not very different from each other on a genomic scale (i.e. separating branch nodes between them were short and/or not supported by bootstrapping). Very different patterns emerged for the Scandinavian samples, however: instead of clear subclades, genetic distances between Bodø and Varberg increased more gradually. Also, differences between individuals within populations were in many cases smaller than in the East Atlantic clade, and especially so in Varberg.

**Fig. 4:**
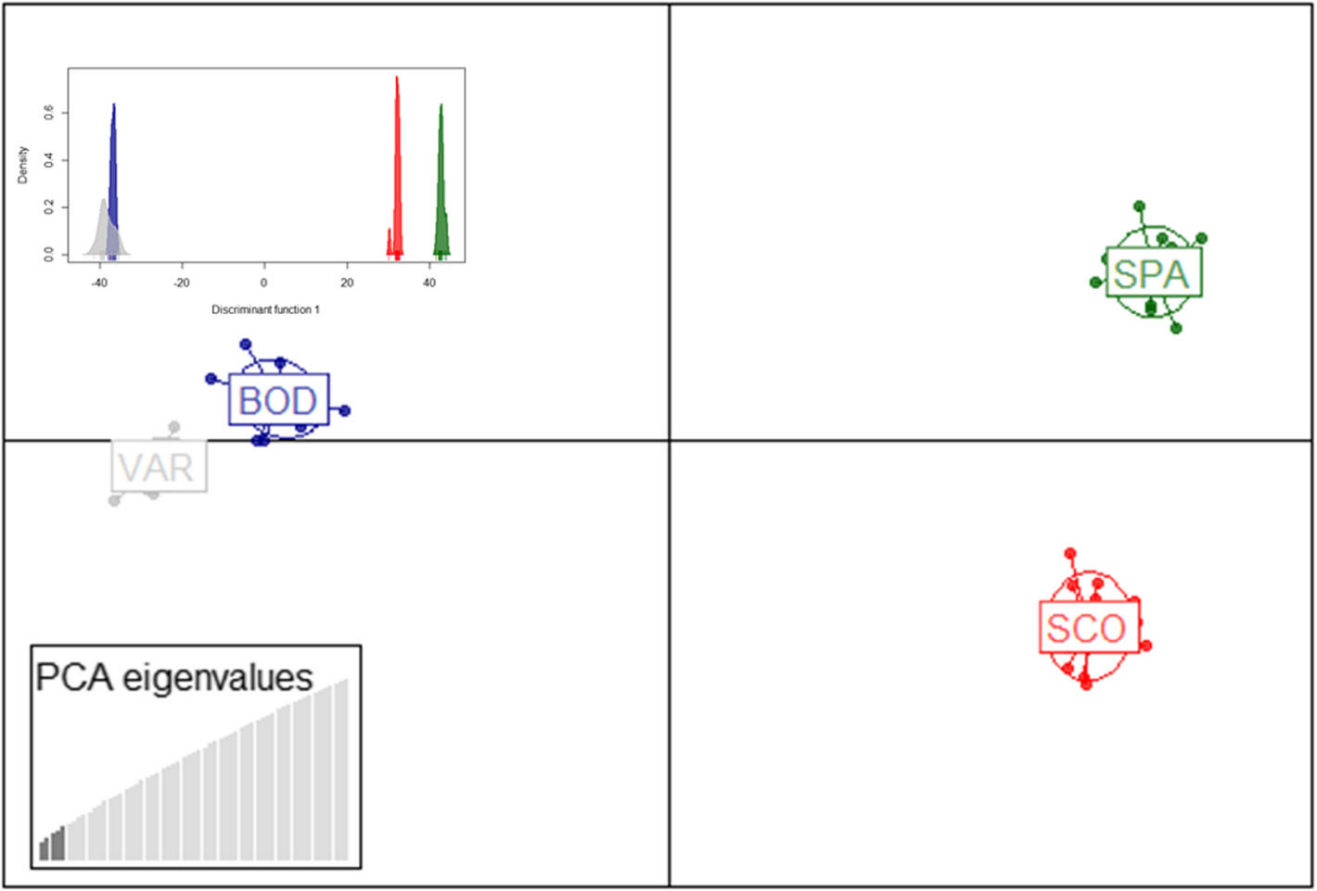
DAPC plot with 33 k data. Optimized number of PCs (5; see Fig. S1), was used together with 3 (main figure) or 1 discriminant function(s) (small figure on upper left corner). Colour coding for populations: Varberg = grey, Bodø = blue, Scotland = red, and Spain = green

Population clustering analysis based on discriminant analysis of principal components (Fig. 4) showed a very similar pattern to the analyses described above; largest separation between north and south, clear distinction between Scotland and Spain, as well as Scandinavian populations rather close each other but still as distinguishable clusters, and Bodø closer to East Atlantic populations than Varberg. All individuals were re-assigned back to their populations of origin with no signs of admixture (Figs. S3a and b).

Tests for evidence of selection indicated the presence of many outliers in both datasets with both approaches (see Figs. S4a-b, 5a-d and 6a-d). For the whole dataset including all four populations, 1379 SNPs (4.2%) were suggested as outliers with PCadapt, and 1209 (3.7%) BayeScan. Four hundred thirteen of the selected loci displayed concordance between methods. When considering Scandinavian populations only, 372 SNPs (1.2%) deviated from expectations under neutrality according to PCadapt analysis and 203 (0.7%) with BayeScan. Eighty-three of these loci displayed concordance between the methods. Despite the applied distance filter of at least 1000 bp between SNPs, some contigs (0.9%) contained more than one or two outlier SNPs (Supp. Table 5); possibly as a sign of stronger selection affecting many SNPs along the same sequence. Based on these results, it is possible that selection plays a role in population differentiation on large geographic scale as well as within Scandinavia. However, with no annotated reference genome available for goldsinny wrasse (or any closely-related species), the possible biological significance related to the detected outlier SNPs remains to be investigated in the future.

### Validation of selected 173 SNPs to separate Scandinavian populations

A total of 231 SNPs was pre-selected as possibly diagnostic based on the estimated high divergence from sequence data between the two Scandinavian populations (Table S1a). Of these, 173 (74.9%) produced reliable genotypes with the used genotyping platform. These loci provided independent genetic information, i.e. no significant linkage between them after FDR correction was found (Fig. S7). Mean expected heterozygosity for the loci was high (0.387), and rather similar for both populations (Table 1; for locus-wise information, see Table S3), but observed heterozygosities were much lower showing significant mean overall heterozygote deficit (*F*_*IS*_ *=* 0.228; 95% CI 0.183–0.272), as well as in both populations separately (Varberg: *F*_*IS*_ *=* 0.196; 95% CI 0.131–0.232 and Bodø: *F*_*IS*_ *=* 0.263; 95% CI 0.222–0.279). Many of the used loci showed significant deviations from HWE after FDR correction: 37 loci (21.4%) in Varberg, and 46 (26.6%) in Bodø. Closer inspection by eye of the deviating loci revealed that for many (but not all) non-HWE loci alternate alleles were predominant in south and north (data not shown).

Using the reduced set of 173 putatively diagnostic SNP markers, the mean *F*_*ST*_ between Varberg and Bodø was 0.065 (Table 2), almost three times higher than the average estimated with the 33 k dataset. However, compared with the used pre-selection criterion of the SNPs (*F*_*ST*_ ≥ ~ 0.15 based on sequence data from 30 individuals), the observed divergence from 94 genotypes for these same loci was surprisingly low (Table S3), and the correlation between these two methods was weak even when *F*_*ST*_ was calculated from an identical set of individuals and markers (Fig. S8a; *R*^*2*^ = 0.028, *p* = 0.029). Locus-wise *F*_*ST*_ measurements between the populations showed that only 69 of the 173 loci (39.9%) were significantly differentiated (*p* < 0.05; not corrected for multiple comparisons; Table S3) and thus contributed to the overall divergence (Fig. S9). If considering only these 69 loci with significant divergence between the populations, the mean *F*_*ST*_ was 0.140. The discrepancy between the methods reduced when a second step of individual-based quality filtering was applied: The observed frequencies of matching, missing and non-matching genotypes were respectively 64, 9 and 27% for the initial set of 173 SNPs, whereas it was 76, 6 and 18% for the set of 74 SNPs. Similarly, the correlation between obtained *F*_*ST*_ from the sequence data and genotype data passed from *R*^*2*^ = 0.028 with the 173 initial SNPs to *R*^*2*^ = 0.344 (*p* < 0.001) with the set of 74 SNPs (Figs. S8b-c). The better reproducibility of 74 loci did not hold, however, when the larger dataset of 47 × 2 fish was compared with the sequence data (*R*^*2*^ = 0.047, *p* = 0.064; Fig. S8d). Locus-wise *F*_*ST*_s derived from small (15 × 2) and large (47 × 2) datasets derived from genotyping were strongly and significantly correlated (for 173 loci, Fig. S8e: *R*^*2*^ = 0.391, *p* < 0.001; for 74 loci, Fig. S8f; *R*^*2*^ = 0.500, *p* < 0.001) suggesting that the used genotyping method gives more consistent estimates, and that the observed discrepancy likely stems from sequences. Possible explanations and implications of discrepancies between methods are discussed below.

**Fig. 5:**
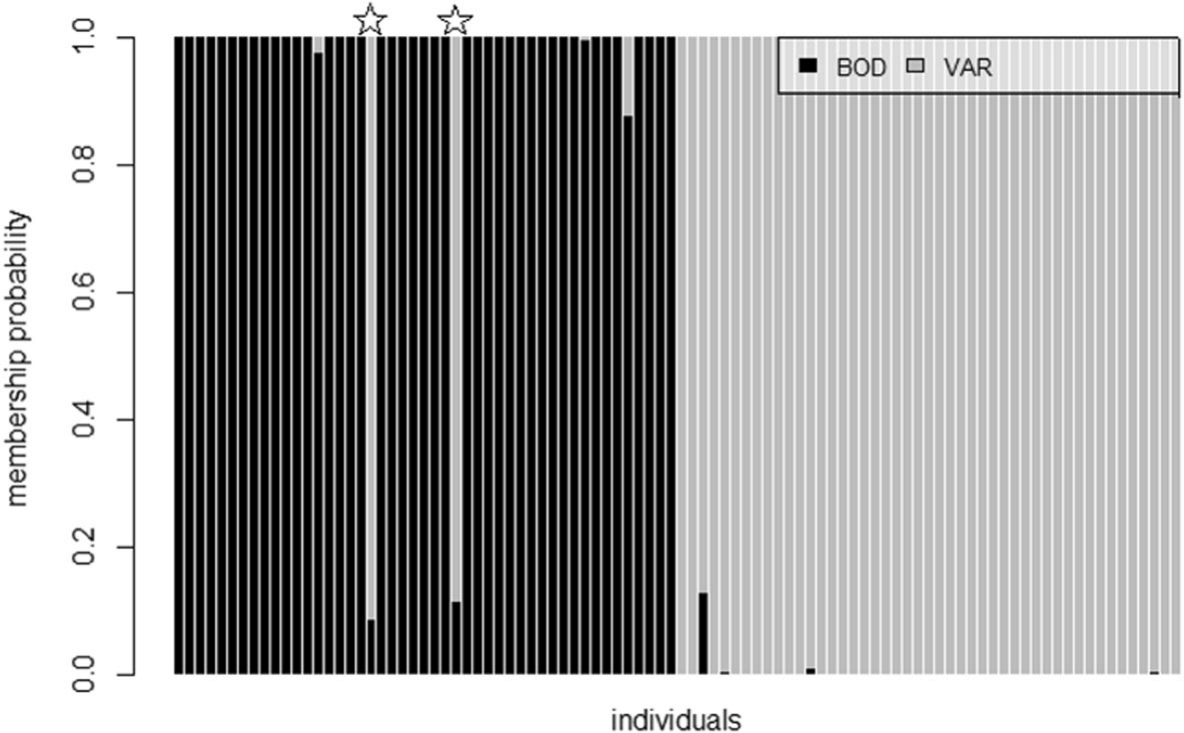
Compo plot of membership probability of the genotyped 94 individuals based on 173 loci. Individuals sampled in Bodø but assigned strongly to Varberg are marked with stars

**Fig. 6:**
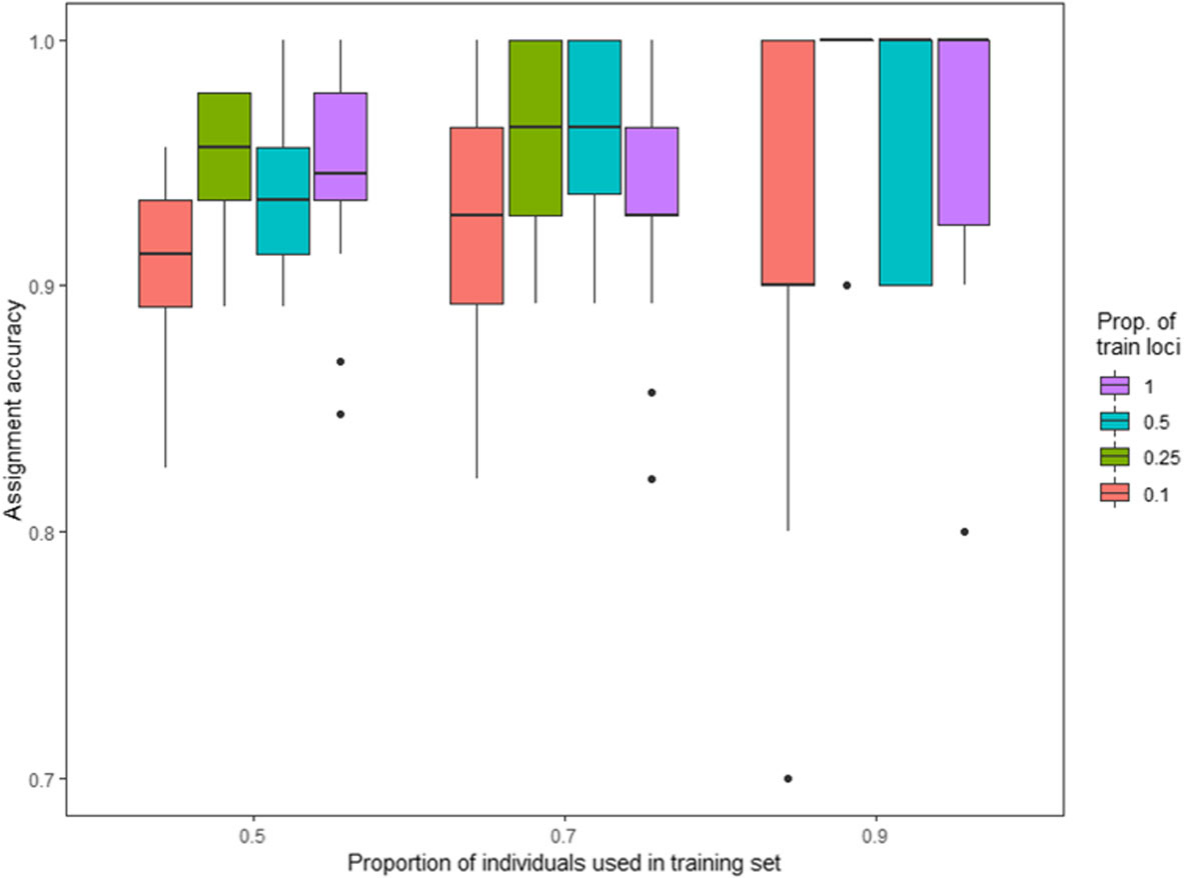
Overall assignment accuracies estimated via Monte-Carlo cross-validation. Three levels of training individuals (50, 70 and 90% of individuals from both populations, on x-axis) were crossed by four levels of training loci (top 10, 25 and 50% highest *F*_*ST*_ loci and all loci in color-coded boxes) by 30 resampling events. Results divided by populations are given in Fig.S10

The 173 SNPs provided a high level of accuracy in assigning individuals back to their populations of origin. DAPC-based analysis gave a mean assignment probability of 97.9% (Fig. 5); 100% for samples originating from Varberg and 95.7% for samples from Bodø. Two fish (4.3%) sampled in Bodø had high membership probability (≥0.9) into the southern population, but otherwise individuals showed very little admixture. Evaluation of the assignment across 360 tests from Monte-Carlo cross-validation showed also high accuracy. Mean assignment for Bodø was 0.95 (±0.07 S.D.) and for Varberg 0.94 (±0.08). Assignment accuracy was in general high (~ 90% or higher), but varied somewhat depending on the number of loci used and samples analyzed (Fig. 6).

## Discussion

This is the first population-genomic study of the goldsinny wrasse, a marine fish that has recently reached high economic value and harvest exploitation due to its importance as a cleaner-fish in commercial salmonid aquaculture throughout the North Atlantic. Based on the production of a de novo assembly, whole genome re-sequencing and identification of SNPs, we demonstrated that goldsinny wrasse displays a highly polymorphic genome of ~600Mbp, and substantial population genomic structure throughout its native range. We were also able to identify and validate sub-sets of loci that were collectively diagnostic between samples of goldsinny wrasse from northern Norway and Sweden. These SNPs now provide an efficient tool for investigating inadvertent translocations and possible non-native introgression of wrasse from the harvest and export regions in southern Norway and western Sweden to the import aquaculture region in mid-Norway.

Teleost fish genomes are known to vary a lot in size (see e.g. [67]), and thus generalizations of genomes are hard to make. However, the two other species in the *Labridae* family that are also used as cleaner fish in commercial salmonid aquaculture, have recently been assembled and their genomes were 805 Mbp for the ballan wrasse [63] and 614 Mbp for the corkwing wrasse [70]. Thus, the goldsinny wrasse genome is rather similar in size with its closest relatives that have been studied. From an evolutionary perspective, however, these cleaner wrasses are distantly related. It has been estimated that the basal split between the genus Ctenolabrus (including goldsinny wrasse) and the other two genera (Labrus with ballan wrasse and Symphodus with corkwing wrasse) occurred at least 14 MYA [42], so their cross-species usability in e.g. reference-assisted genome assembly is likely limited. Genome-level compatibility between the species was tested in connection with this study (data not shown) by aligning the used 1222 > 20 k contigs against the available reference genomes in the GenBank® for both species (ballan wrasse; https://www.ncbi.nlm.nih.gov/assembly/GCF_900080235.1, corkwing wrasse; https://www.ncbi.nlm.nih.gov/assembly/GCA_002819105.1). Sequence uniformity was on average 71.68% between goldsinny and corkwing wrasse, and 77.87% between goldsinny and ballan wrasse. There was very wide variability in the similarity between pairs of sequences, however, ranging from ~ 10 to > 90% match between sequences suggesting largely differing genomic compositions between the species.

The high genomic variability revealed here (Fig. 2 showing bimodal kmer distribution and estimated ~ 10 million variable sites in the reference) is consistent with an abundant marine fish covering large distribution area and with partly pelagic eggs (Hilldén estimated in 1984 that ~ 10% of the eggs float and may be transported by currents). This is because large (effective) population size and/or high connectivity between populations are known to be positively correlated with genetic diversity (e.g. [1, 32])

High genetic variability was also observed in all the four studied populations, with an average of 30% heterozygosity or more (Table 1). This is well in line with the detected levels of variability in our previous study (0.349–0.367; see Table 2 in [47]) employing a set of 36 SNPs. Due to the protocol implemented here to discover polymorphic SNPs however, it is likely that rare allele variants were not included, thus inflating the observed mean level of variability, and that some part of the observed differences in variability between populations are due to sampling biases (see e.g. [58, 68]). Our de novo reference was assembled from a fish caught in Varberg, a population which also displayed the highest heterozygosity levels (Table 1), and contrary to other populations (which showed slight but significant heterozygote deficiencies), significant general heterozygote excess on the genomic scale. To rule out if biological processes, such as non-random mating and gene flow [2], account for these observed general genetic patterns and the differences observed between the populations, population studies including more samples, would be needed [30]. Interestingly, in our previous study [47] with 36 SNPs and 14 microsatellites, the same set of samples from Varberg (*N* = 94) showed significant heterozygosity deficiency with both marker types. These 50 markers were developed from ddRAD sequences without any reference genome [48] using four other populations than Varberg, implying that the deviant heterozygosity pattern observed here for Varberg, could be a technical artefact.

Results from analyses using the genome wide panel of 33 k SNPs revealed that goldsinny wrasse populations from different parts of the species distribution area (Fig. S1) are clearly and significantly differentiated from each other. In fact, the observed level of pairwise divergence (*F*_*ST*_ = 0.015–0.092; Table 2) is somewhat higher than in our previous study with 36 SNPs (*F*_*ST*_ = 0.013–0.049; see Table 3 in [47]), and in general quite high compared with many other marine organisms with pelagic life stages (e.g. [8, 16, 24, 97]). Divergence between goldsinny wrasse populations is not necessarily due to geographic distance and restricted gene flow alone. A notable proportion, ~ 1% within Scandinavia and ~ 4% globally, of the diverged SNPs were identified as outliers likely under selection (Figs. S4–6). However, GenBank® nucleotide searches for the top-outlier SNPs did not retrieve any hits. Thus, their possible biological role (see e.g. [28, 74]) remains to be studied in the future when an annotated genomic reference, and/or genetic linkage map is available.

The constructed individual-based genomic phylogeny tree (Fig. 3) suggests that Scottish and Spanish populations are closer to North than South Scandinavian populations, and thus, that goldsinny wrasse in South-Scandinavia might stem from populations higher north along the coastline. This result is somewhat surprising because even though shortest oceanographic distances between the Scottish and both Scandinavian populations are roughly similar (Fig. 1), direct gene flow across the North Sea is highly unlikely [47], and thus South Scandinavia is more reachable along the coastline. Moreover, within Scandinavia, passive drift – and thus possibly also gene flow is predominantly unidirectional from south to north along the Norwegian Coastal Current [47]. Considering a longer-time evolutionary context could elucidate the result, however. After the last ice age, the Scandinavian Ice Sheet retreated gradually around 20,000–10,000 years ago, starting from the Danish and Norwegian west coasts [46]. Depending on colonization routes and modes of dispersal, it is possible that many fish species first recolonized the southwestern corner of the present-day Norway, and spread from there north and/or south when more of the coastline re-emerged. Such population history of step-wise colonization would explain the observed phylogeny for goldsinny wrasse (see also e.g. [4, 40]). In a recent study, Mattingsdal et al. [71] showed that the current-day population structure of corkwing wrasse in Scandinavia characterized by a substantial division between western and southern Scandinavia can mainly be explained with past demographic events followed by reproductive isolation and genetic drift. Similar colonization history was newly suggested for ballan wrasse [84]. Unlike corkwing and ballan wrasse for which Scandinavian western and southern populations are quite isolated (but see [71] proving some gene flow across the genetic break), the Scandinavian goldsinny population is characterized by extensive gene flow following oceanic currents, weak general population structure, increasing genetic divergence with oceanographic distance (i.e. isolation-by-distance, IBD), and with no clear breaks [47]. We conclude that the present-day population structure of goldsinny wrasse is therefore likely to represent a combination of past demographic processes shaping structures on larger scale, and dispersal mainly between nearby areas leading to limited gene flow and IBD. Even though demographic history and genetic drift would be the dominant evolutionary processes shaping the contemporary population patterns, other factors like isolation-by-adaptation, polygenic selection, and human interference may also play an important role and require further study.

Genome-wide datasets represent powerful tools with which to detect subtle population genetic differentiations [8, 16, 73], patterns of selection [11, 79, 94], as well as other selective responses such as genome re-arrangements [55, 90]. However, at the present, genomic analysis does not provide a cost-effective approach for many fisheries management purposes [69] where screening many individuals with fewer targeted or diagnostic loci is often more feasible approach [23, 34, 49, 66]. The panel of 173 SNPs developed in the present study to separate Scandinavian goldsinny wrasse populations from north and south proved to have high assignment accuracy. Compared with the random genomic panel with an average *F*_*ST*_ of 0.024 between the populations (and 0.017 measured with 36 SNPs in [47]), the putatively diagnostic loci developed here showed almost threefold average differences in divergence (*F*_*ST*_ = 0.065). The divergence based on sequence data was much higher, however (Table S1a), and there were large differences between loci between the expected and observed level of *F*_*ST*_ (Table S3, Fig. S8a). These discrepancies are probably due to different reasons: First, the sequence data consisted only of 15 fish per population compared with 47 per population genotyped for validation. Small sample sizes are adequate for estimating general genetic differentiation between populations on a genomic level [98], but for any specific locus likely much more sensitive to biases. Also, the SNP selection itself can introduce systematic upward bias when loci are screened for maximum divergence for population assignment without appropriate cross-validation procedures [5]. Furthermore, NGS methods are prone to diverse errors (e.g. [68, 78]) also likely partly accounting for the lower than expected observed divergence, and lack of correlation between the two methods. Post hoc analysis of the selected SNPs revealed that individual filtering of SNPs based on coverage and quality could improve reproducibility between methods (Fig. S8a-c). This is in line with the earlier observation that variance in read coverage between individuals and between loci in the same individual introduce biases [68]. However, based on results from this study, larger sample sizes are likely also needed in search of reliable outliers between populations (Fig. S8c cf. S8d; see also [5]). Using another sequencing approach, such as some reduced representation method, could have given us larger samples per population and thus higher precision of observed allele frequencies. On the other hand, this in turn would have come on the expense of genomic coverage, and thus possibility to detect (any) adaptive outliers [45], and could also introduce other type of errors and biases (see e.g. [38, 68, 99])

From the individual assignment aspect, it does not matter if allelic differences between populations have arisen from neutral or adaptive evolutionary processes. However, SNPs associated with adaptive processes, and potentially local adaptations are more likely to remain divergent even in the face of moderate or even high levels of gene flow (e.g. [27]). Such SNPs are thus biologically and from a management perspective important. In this study, two individuals caught in Bodø (4.2%; Fig. 5) were strongly assigned to the southern population (Varberg). Natural, direct gene flow between so distant locations is very unlikely [47] raising a question of possible translocations of wrasse from aquaculture. Faust et al. [29] showed transported wrasses can survive and hybridize with local fish. In their study, of the sampled 40 wild corkwing wrasses in Flatanger area in mid-Norway, 2 were translocated fish, 1 first-generation hybrid and 12 possible second-generation hybrids. Considering that corkwing and goldsinny wrasse are by far the most abundant catches (between 8 to 12 million each annually during the last 5 years; Directorate of Fisheries; www.fiskeridir.no), and transported in great numbers, human-mediated gene flow and hybridization is a plausible sequence of events. But to decide whether these two fish are indeed translocated fish from south and/or hybrids between translocated and local fish, and how frequent such introgression would be in recipient areas in mid-Norway, the population structure and composition between these two sampling localities must be investigated in detail using the diagnostic SNPs developed here.

## Conclusions

In this study, full-genome sequencing was used to produce a de novo assembly and thereafter investigate the population genomic structure of goldsinny wrasse in four geographically distinct locations. This is an economically important marine fish that is subjected to high harvest rates in some regions, and translocation to fish farms in other regions where it is used to delouse farmed salmonids. We demonstrated that the goldsinny wrasse genome, ~600Mbp in size, possesses high level of genetic variation, and that populations on different sides of the species distribution area are genetically significantly differentiated. Based on the conducted screening of outlier loci, some of the genetic differences observed among these populations are likely to be associated with functional divergence. For the two Scandinavian populations, we tested and evaluated a panel of putatively diagnostic SNP loci that proved to provide high resolution. Such markers now provide a tool with which to study human-mediated translocation between geographic areas through aquaculture practice.

## Methods

In this study, new genetic markers for goldsinny wrasse were developed and validated by: I) sequencing one goldsinny wrasse sample with high coverage to build a genomic reference de novo, II) selecting the longest contigs from the assembly, and III) mapping individual reads from 60 fish from four populations (15 from each) against the reference contigs to call SNPs. A random subset of the high-quality SNPs obtained (1 SNP/~kbp, in total 33,235), were then used to, IV) run population genetic analyses to explore basic population parameters, and to characterize genetic differentiation among the populations as well as to look for signs of selection. Finally, V) a set of 173 candidate SNP loci showing large divergence between northern and southern Scandinavian populations was selected and validated using Agena MassARRAY® iPLEX platform with an additional set of samples (*N* = 94).

### Samples and sequencing

Sixty goldsinny wrasse from four locations were used to search for and develop SNP markers against the reference contigs from one individual. Sixteen individuals (including a single fish used to build a reference genome) were collected from Varberg (VAR) in south-western Sweden, 15 from Bodø (BOD) in Northern Norway, 15 from Isle of Mull in Scotland, UK (SCO), and 15 from Galicia region (GAL) in north-western Spain (Fig. 1). These samples were collected in 2014–2016, and represent a subset of the samples used in a previous study of population genetic structure [47]. Samples were collected in compliance with EU Directive 2010/63/EU, and the national legislations in each country. Fish were killed upon catch and samples were taken immediately or killed and whole fish stored frozen until sampling in laboratory facilities. Details on data collection and DNA extraction are provided in [47]. Samples were selected I) to cover the species’ north-eastern Atlantic distribution area, II) to represent genetically most diverged populations from this area (based on results in [47]), and III) being of good DNA quality and quantity. DNA quality was assessed by running samples on a 1% agarose gel, measuring their absorbance by a Nanodrop spectrophotometer, and by estimating DNA quantity with Qubit Quant-iT kit from Invitrogen. Superior samples (size > 10,000 bp, Abs_260/280_ = 1.8–2.0 and Abs_260/230_ = 1.8–2.4) from each population were selected for sequencing (see below). 4 μg of DNA from the selected reference individual (*VAR-ref-77*; sex unknown) and 2.5 μg from each selected individual from the four populations were sent to the Norwegian Sequencing Centre (NSC) for sequencing. At the NSC, Illumina TruSeq adapter ligation was used to construct libraries, DNA fragmented to 300 bp target size, and barcoded to enable individual identification. Sequencing was done using an Illumina HiSeq X instrument producing 2 × 150 bp paired-end (PE) reads. The reference individual was run in a single lane, whereas the rest of the samples (60) were pooled in four lanes according to their origin (15 individuals/lane, all individual barcoded). Each lane produced between 302 and 316 GB of data as FASTQ files; for the reference individual, we obtained 451.1 million read pairs, and for pooled population samples 455.8–474.5 million read pairs per lane (on average 30.7 ± 12.9 × 10^6^ read pairs per individual, range 16.0–102.4 × 10^6^).

### Building and evaluating the de novo reference

Read quality was checked with FastQC [6], and followed by trimming of sequences with Trimmomatic (v. 0.32 [15];) where adapter contaminants and low-quality reads were removed using the following setting: phred score ≥ 33, leading and trailing low-quality bases removed, scanning in 4 bp windows and cutting when the average quality per base drops below 15, minimum length for a sequence to be retained = 36 bp. Only correctly paired, high-quality reads were retained for the next phase, and consisted of 92.5% (~ 417 million) of the initial raw reads. Genome size and average sequencing coverage was estimated using K-mer statistics with a K-mer size of 32 and software *kmx* (K-Mer indeXing; available at: https://github.com/ketil-malde/kmx). Expectation-maximization was used to fit a negative binomial distribution to the error K-mers, and Poisson distributions for haploid, diploid, and repeat K-mers.

To build a de novo reference, we used SOAPdenovo2 (v.2.0.4 [64];) with default parameter values and increasing K-mer sizes (bp) from 43 to 123 (every tenth), and with the assembler’s possible maximum value of 127. Of these 10 assemblies, the assembly with K-mer size 127 produced the longest continuous scaffolds (based on scaffold length and *N50*), and was selected as our genomic reference. The reference quality was assessed based on contig lengths and their total amount (i.e. fragmentation), content of core genes (i.e. how many percent of ray-finned fish core genes were found using BUSCO (Benchmarking Universal Single-Copy Orthologs, v. 2.0.1 [87];)), and by checking the mapping success of each individual against the reference (as % of reads mapped).

### SNP calling, selection and validation

Individual reads from all 60 fish were aligned against the de novo reference genome using *mem* method in BWA (v. 0.7.5a [61];), using default parameters. Sorting, indexing and variant calling was done with SAMtools (v.0.1.19 [62]), and followed by filtering with BCFtools (v. 0.1.17 [25];). Supplementary alignments were removed, and due to computational constraints, only reference scaffolds > 20 kbp were included. The *vcfR* package [56] in *R* (version 3.2.2 [81];) was used to scrutinize quality and quantity of the aligned sequences, and the following filtering criteria (per population) was chosen for any SNP to be accepted: min_QUAL = 600, min_DP = 20, max_DP = 999, min_MQ = 0, and max_MQ = 90. Due to observed discrepancies between the expected genotypes from mapping data and the observed genotypes from the genotyping platform (see 2.3 in Results), an additional a posteriori step of SNP selection was implemented. The output of the mapping was re-examined, and the average of the individual phred-scaled quality score was calculated for each of the 173 SNPs within Bodo and Varberg samples separately. The average individual phred-scaled mapping quality ranged from 0 to 100 in the Bodo sample and from 0 to 80 in the Varberg sample. SNPs with both average quality larger than 50 in Bodo sample and larger than 40 in Varberg samples, were selected as a subset of the 74 most robust markers.

Two separate data sets were created to: 1) Study population genomic patterns and divergence among the populations. For this, a random subset of the high-quality SNPs was selected along the longest scaffolds so that the physical distance between them was ≥1000 bp. This resulted in a data set of 33,866 SNPs. 2) Select putatively diagnostic SNPs for pair-wise separation of the four populations. To do this, the SNPs showing highest pairwise *Fst* between each population pair were selected from each scaffold. These SNPs were thereafter ranked from the highest to lowest pairwise *F*_*ST*_ (for each pair separately) down to a value of ~ 0.15, leaving a few hundred candidate loci per pair (~ 450–850; Tables S1a-f). To validate the SNPs that separated the Northern and Southern Scandinavian populations, 231 of the top-SNPs were selected and organized into eight multiplex groups (28–30 SNPs in each; Supplementary Table 2) using the MassARRAY® Typer 4.0 Assay Design software (Agena Bioscience). Putatively diagnostic SNPs for other pair-wise comparisons were developed (Supplementary Tables 1b-f), but not validated in this study. Genotyping to validate the diagnostic SNPs developed between the northern and southern Scandinavian samples was performed on a MassARRAY® Typer 4.0 Analyser (Agena Bioscience). Only loci that were polymorphic and produced clear clustering patterns, were selected leaving 173 SNPs. Forty-seven samples from Bodø in Northern Norway and Varberg on the Swedish West coast representing roughly the current edges of the species’ distribution area in Scandinavia, were genotyped and analysed.

### Population analyses of genome wide data

First, 609 of the 33,866 SNPs showing no or very little variation (MAF < 0.01) were removed. Remaining loci were checked for possible deviations in Hardy-Weinberg equilibrium (HWE) using *pegas* package v. 0.11 [76] in *R*. 22 SNPs not in HWE after False Discovery Rate correction (FDR [9];) in at least two of the studied populations, were removed from the final dataset (*N*_*loci*_ = 33,235). Linkage disequilibrium was tested by computing the correlation coefficient (r^2^) between all pairs of loci that were physically linked on the same scaffold. In addition, a random set of 100,000 pairs of loci was sampled across scaffold, to produce a reference distribution of r^2^ values without physical linkage. The *R* package *hierfstat* (v. 0.04–28 [37];) was used to calculate gene diversity (*H*_*S*_), and observed heterozygosity (*H*_*o*_) and overall *F*_*IS*_ within populations. Pairwise *F*_*ST*_ values [96] were estimated for each population pair and over all samples using the same package, and their statistical significance was determined comparing the observed values with 95% confidence limits determined by 1000 bootstrap repeats. For examination of genetic relatedness between samples, *R* packages *poppr* (v.2.8.1 [53];) and *ape* (v. 5.2 [77];) were utilized to reconstruct a genetic distance tree based on information from all 33,235 SNPs using UPGMA algorithm and 100 bootstrap replicates to assess branch support.

To investigate population structure further, and test assignment probability of individuals back to populations based on their genotypes, we performed a Discriminant Analysis of Principal Components (*DAPC* [52];) using the R package *adegenet* (v. 2.1.1 [50, 51];). This is a multivariate method useful for exploring separation of groups in large genomic datasets, even when the general level of divergence between populations is low [52]. The method identifies structuring alleles, and maximizes among-group variation by first transforming the genotype data into principal components (PCs), followed by discriminant analysis (DA) to define the groups. The number of clusters (*K*) was determined by the *find.clusters* function with a maximum *K* set to 10. The lowest BIC (Bayesian Information Criterion) value was obtained with *K* = 4. To avoid model overfitting, the optimal number of PCs was determined via α-optimization to be five (Fig. S2), which was then used in the following *DAPC* analysis and to define group membership (i.e. individual assignment probabilities to predefined populations).

To estimate if and to what extent natural selection could explain divergence between populations, the *pcadapt* (v. 3.0.4; [65]) package in *R* was employed to perform a genome scan to detect markers potentially influenced by selection. This was done for the whole dataset, and separately for the Scandinavian samples only. The method first performs a principal component analysis (PCA) to ascertain the underlying population structure, and thus allows uncertainty of origin and admixed individuals. Based on obtained ‘*scree plots*’ from the PCA analysis, the optimal value of PCs was four for the whole dataset (Fig. S5a), and two for the Scandinavian dataset (Fig. S6a), and *K =* 4 and 2, respectively, were used for the subsequent *pcadapt* analyses. SNPs with minor allele frequency below 0.05 were removed from the analysis leaving 32,712 SNPs in the whole and 31,941 SNPs in the Scandinavian datasets. A locus was considered as an outlier if its q-value threshold was below 0.1. Q-value estimation includes FDR and adjust *p*-values accordingly. Q-values were determined by *qvalue* package (v. 2.12.0 [91];) in *R.* Another selection test, BayeScan (v.2.1 [31];) was run for comparison for both datasets. Default parameter setting was used (prior odds 10, samples size 5000, thinning interval 10,000, pilot runs 20, pilot run length 5000 and additional burn-in 50,000). The decision whether a locus was under selection was based on q values (< 0.05 suggests selection). Possible clustering of multiple (> 2) selected SNPs along same contigs was checked.

### Validation and population analysis using putatively diagnostic loci

All 94 genotyped samples produced reliable genotypes with more than 70% of the used 173 loci and were included in the analyses. Because these SNPs were picked as candidates for separating Scandinavian populations best, neutrality and thus HWE cannot be assumed. It was nevertheless investigated, together with other basic population parameters using same methods and approaches as given for the genome wide data analysis. Moreover, each locus pair was tested for linkage disequilibrium using the *snpStats* (v.; 1.32.0 [19];) package in *R* and visualized with *LDheatmap* (v. 0.99–5 [86];).

As for the genomic dataset, population subdivision and individual assignment was inspected with the *DAPC* package. To evaluate the discriminatory power for the baseline data, an additional Monte-Carlo cross-validation through resampling procedure was run with *R* package *assignPOP* (v. 1.1.4 [18];). The resampling scheme contained 50, 70 and 90% of the individuals from both populations, and top (based on *F*_*ST*_) 10, 25, 50% or all loci. Each resampling event was repeated 30 times.

## Supplementary information

**Additional file 1 : Table S1a-f.** List of highly differentiated SNP loci between populations.

**Additional file 2 : Table S2** Amplification groups and other related information for genotyping of the selected 231 loci to separate Scandinavian populations.

**Additional file 3 : Table S3** Basic genetic information for the 173 SNPs used to analyze Scandinavian populations.

**Additional file 4 : Table S4** Additional information of the 1222 > 20 k scaffolds from which the markers used in this study were acquired.

**Additional file 5 : Table S5** List of scaffolds containing more than two SNPs possibly under selection.

**Additional file 6 : Figure S1.** α-plot for the determination of optimal number of principal components retained in DAPC with 33 k data. **Figure S2.** Distribution of observed heterozygosity for the used 33,235 SNP loci over all loci in all populations (main figure). Locus-wise distribution per population is shown in the upper right corner (small figure). **Figure S3 a) and b).** Assignment plot (a) and compo plot (b) of 60 goldsinny wrasses based on 33 k SNPs. All individuals have very high probability to belong to the same population from where they were sampled. **Figure S4 a) and b).** Selection test result from the BayeScan analysis shown as figure for a) the whole dataset, and b) for the Scandinavian populations only. Loci on the right side of the vertical line are observed outliers, and thus suggested being under selection. **Figure S5 a.-d).** Figures related to selection test for the whole genomic dataset (all four populations) with PCadapt a) Scree plot to determine number of Ks. Because the curve plateaus after K = 4, that was the selected number of clusters. b) Histogram of *p*-values. The excess of small p-values indicates presence of outliers. c) Manhattan plot displays -log_10_ of the p-values. d) Q-Q plot show that many p-values do not follow expected uniform distribution confirming outliers. **Figure S6 a.-d).** Figures related to selection test for the Scandinavian genomic dataset (two populations) with PCadapt. **Figure S7.** Heatmap of linkage between the used 173 SNP loci**. Figure S8a-e.** Expected vs observed *F*_*ST*_ between Scandinavian goldsinny wrasse populations. **Figure S9.** Loading plot showing individual locus contribution of the genetic divergence between the Scandinavian goldsinny wrasse populations. **Figure S10.** Assignment accuracy divided by populations with different proportions of the 173 SNP loci based on Monte Carlo resampling procedure.

## Data Availability

Sequence reads from this study are available through NCBI archive from the following web page: https://www.ncbi.nlm.nih.gov/bioproject/PRJNA508986. The final genotype datasets used in this study are available from the Dryad repository: 10.5061/dryad.rjdfn2z7s . Lists of putative diagnostic SNPs identified in this study are given in Tables S1a-f.

## Abbreviations

bp: base pair
df: degrees of freedom
DNA: Deoxyribonucleic acid
FDR: False discovery rate
HWE: Hardy-Weinberg equilibrium
IBD: Isolation-by-distance
MPA: Marine protected area
MYA: Million years ago
*N*: Number (of)
NGS: Next generation sequencing
SD: Standard deviation
SNP: Single nucleotide polymorphism
