# Supplementary material for: Genome wide analysis reveals genetic divergence between Goldsinny wrasse populations
Source: BMC Genet. 2020 Oct 9;21:118. doi: 10.1186/s12863-020-00921-8 (PMC7547435; doi:10.1186/s12863-020-00921-8)
Supplement: Supplementary file 6 — Additional file 6 : Figure S1. α-plot for the determination of optimal number of principal components retained in DAPC with 33 k data. Figure S2. Distribution of observed heterozygosity for the used 33,235 SNP loci over all loci in all populations (main figure). Locus-wise distribution per population is shown in the upper right corner (small figure). Figure S3 a) and b). Assignment plot (a) and compo plot (b) of 60 goldsinny wrasses based on 33 k SNPs. All individuals have very high probability to belong to the same population from where they were sampled. Figure S4 a) and b). Selection test result from the BayeScan analysis shown as figure for a) the whole dataset, and b) for the Scandinavian populations only. Loci on the right side of the vertical line are observed outliers, and thus suggested being under selection. Figure S5 a.-d). Figures related to selection test for the whole genomic dataset (all four populations) with PCadapt a) Scree plot to determine number of Ks. Because the curve plateaus after K = 4, that was the selected number of clusters. b) Histogram of p-values. The excess of small p-values indicates presence of outliers. c) Manhattan plot displays -log10 of the p-values. d) Q-Q plot show that many p-values do not follow expected uniform distribution confirming outliers. Figure S6 a.-d). Figures related to selection test for the Scandinavian genomic dataset (two populations) with PCadapt. Figure S7. Heatmap of linkage between the used 173 SNP loci. Figure S8a-e. Expected vs observed FST between Scandinavian goldsinny wrasse populations. Figure S9. Loading plot showing individual locus contribution of the genetic divergence between the Scandinavian goldsinny wrasse populations. Figure S10. Assignment accuracy divided by populations with different proportions of the 173 SNP loci based on Monte Carlo resampling procedure. [file 12863_2020_921_MOESM6_ESM.docx]

**Supplementary Figures (Figs. S1-10)**

For Jansson *et al*. “Genome wide analysis reveals genetic divergence between Goldsinny wrasse populations”

**
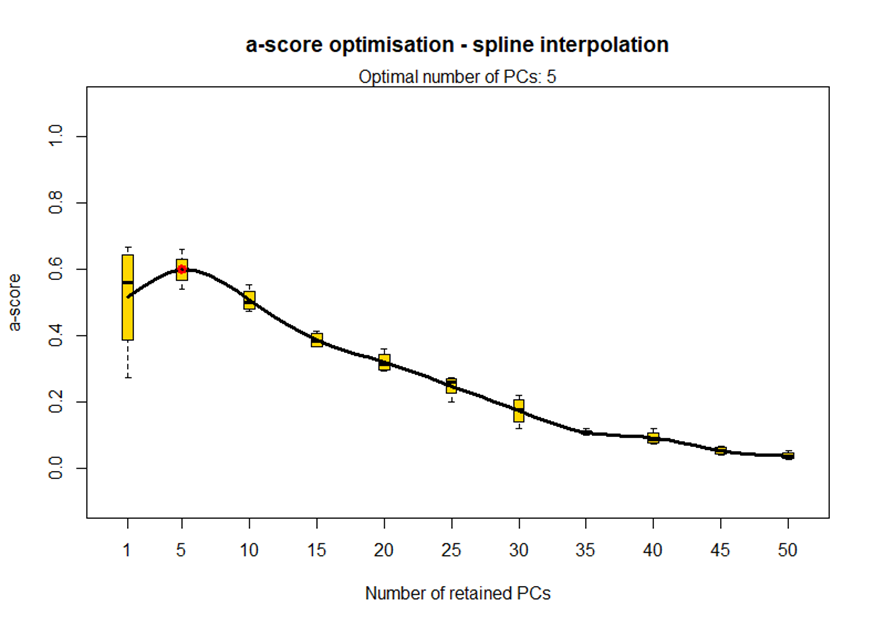
**

**Supplementary Figure 1.** α-plot for the determination of optimal number of principal components retained in DAPC for the 33k SNP dataset

**
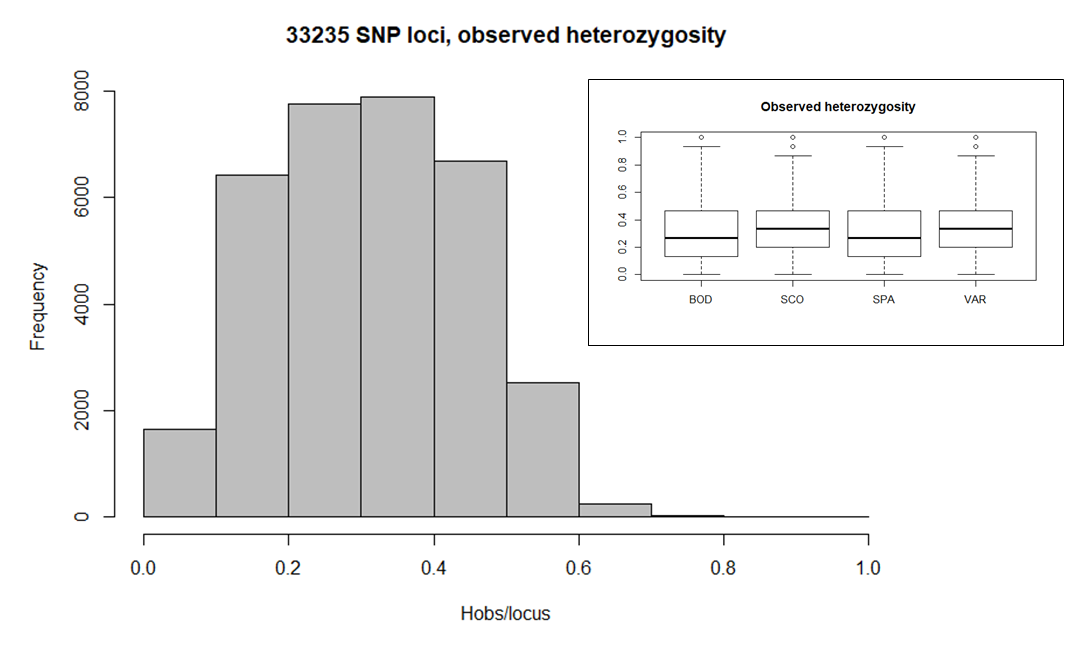
**

**Supplementary Figure 2.** Distribution of observed heterozygosity for the used 33235 SNP loci over all loci in all populations (main figure). Locus-wise distribution per population is shown in the upper right corner (small figure).

**
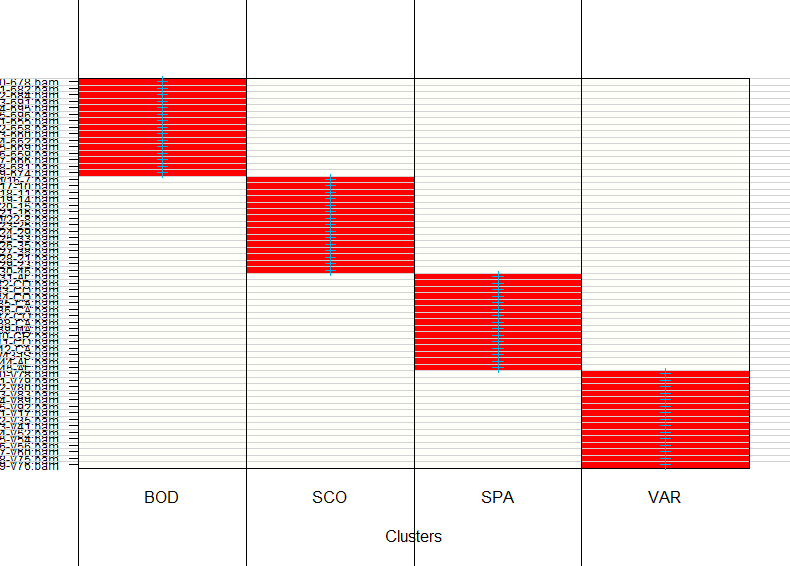
**

**
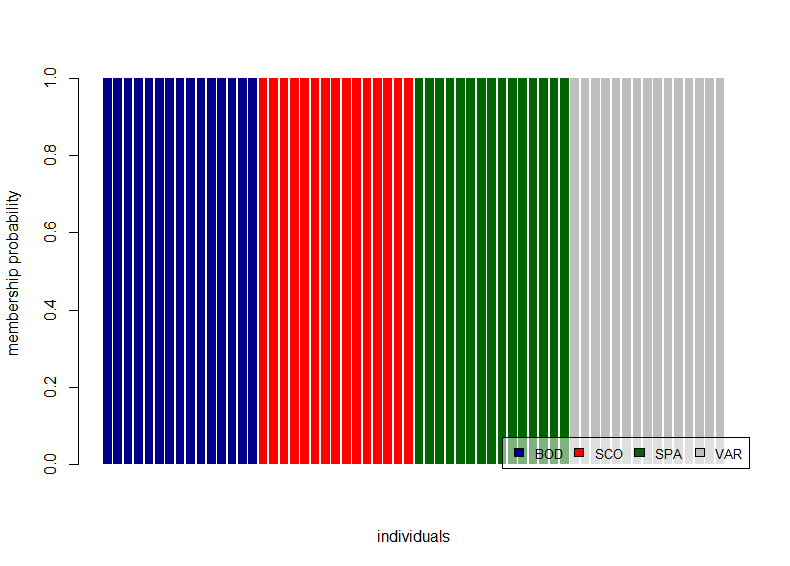
**

**Supplementary Figure 3 a) and b)** Assignment plot (a) and compoplot (b) of 60 goldsinny wrasses based on 33k SNPs. All individuals have very high probability to belong to the same population from where they were sampled.

**
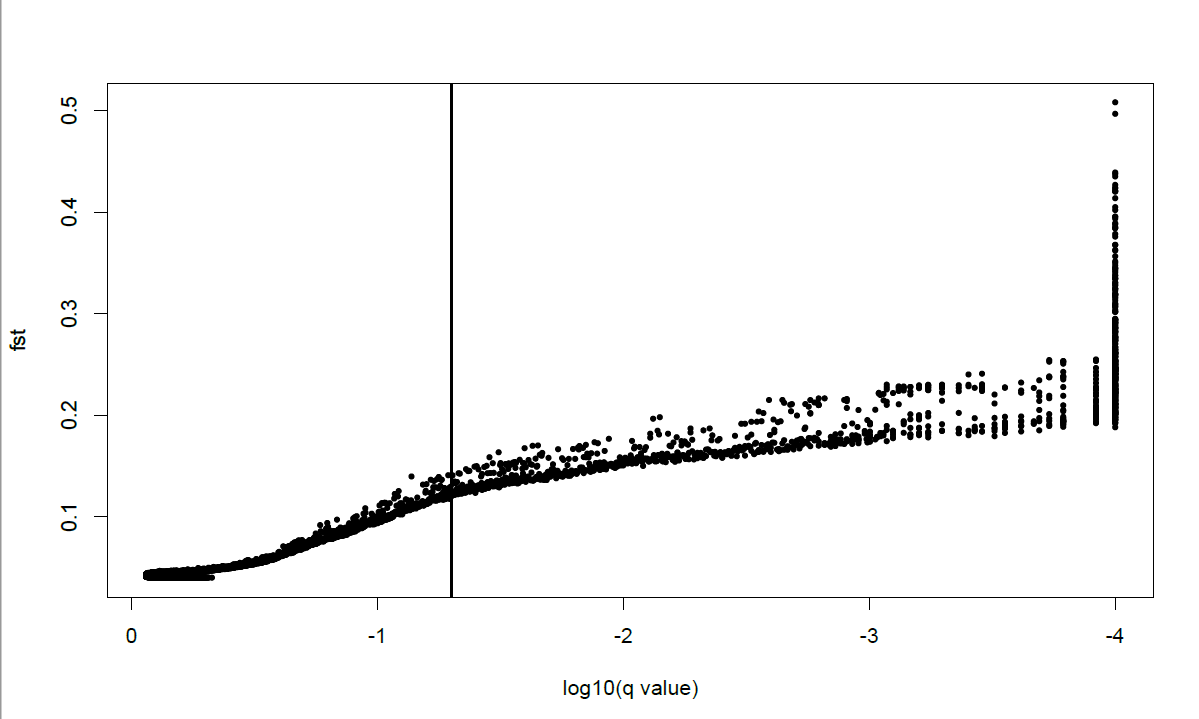
**

**
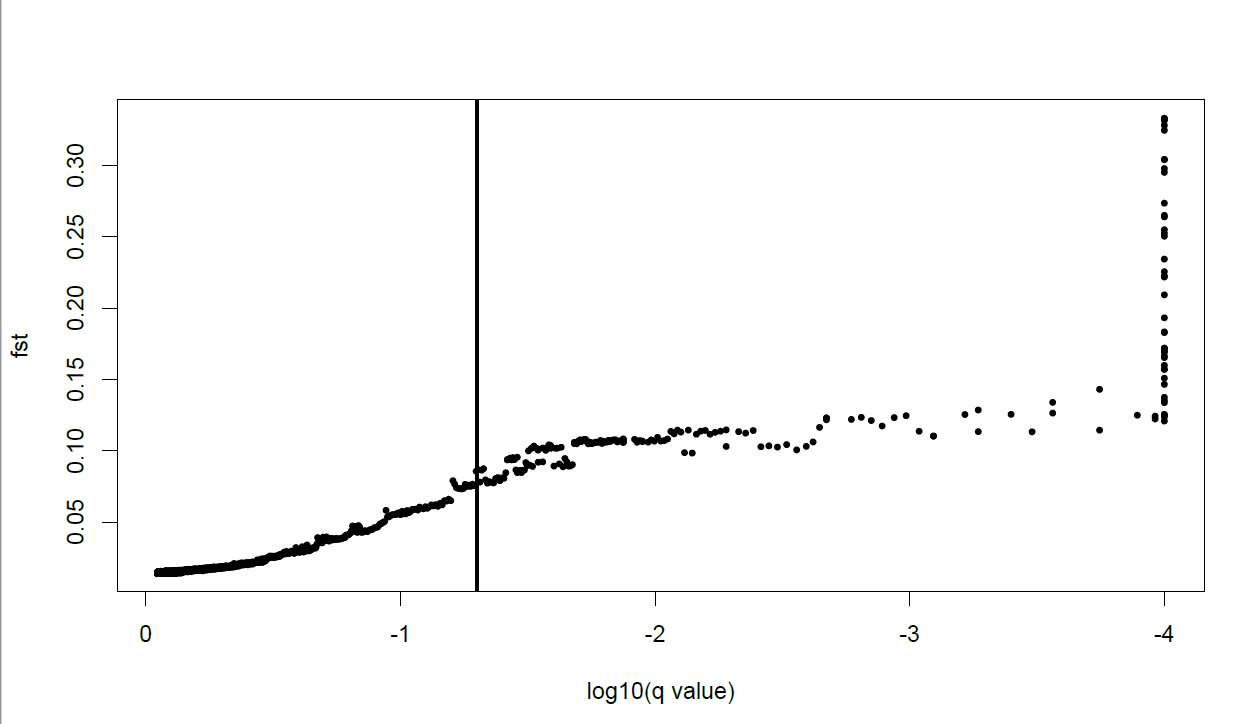
**

**Supplementary Figure 4a-b).** Selection test result shown as figure for a) the whole dataset, and b) for the Scandinavian populations only. Loci shown on the right side of the vertical line are outliers, and thus suggested being under selection. There are in total 1209 outliers in the whole dataset, and 203 within Scandinavian populations.

**
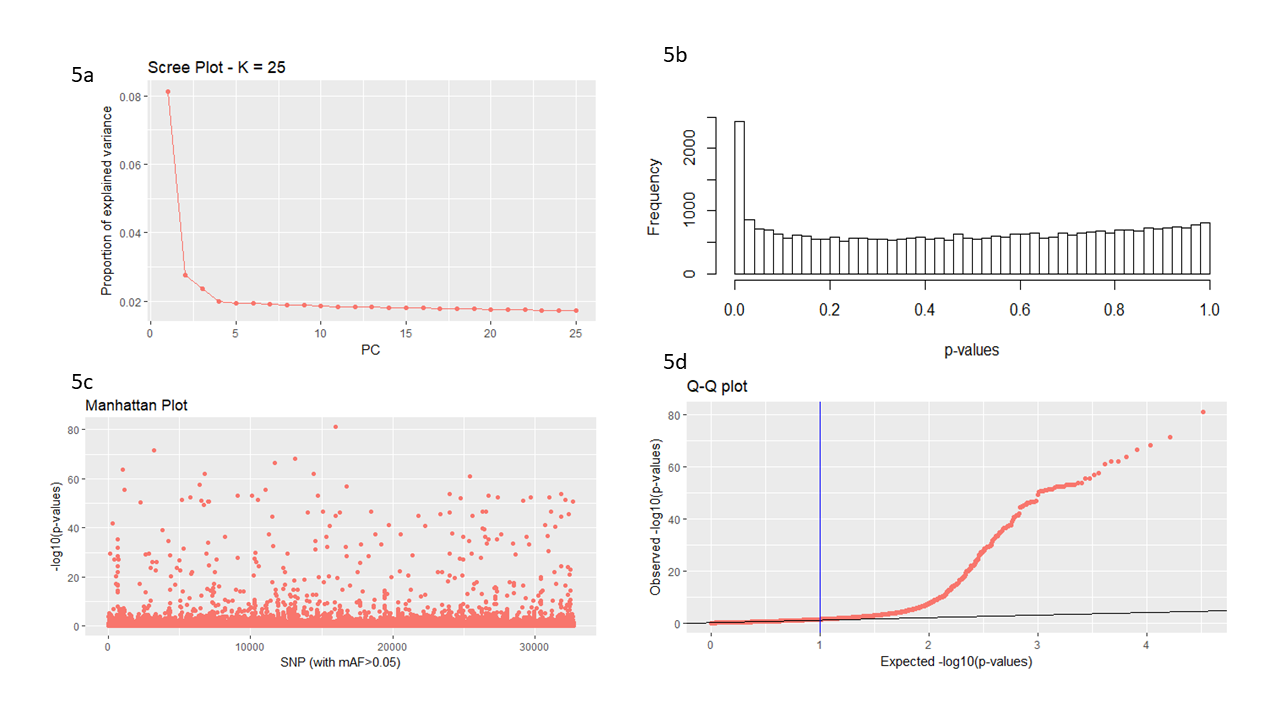
**

**Supplementary Figures 5a-d) Figures related to selection test for the whole genomic dataset (all four populations) with PCadapt** a) Scree plot to determine number of Ks. Because the curve plateaus after K=4, that was the selected number of clusters. b) Histogram of p-values. The excess of small p-values indicates presence of outliers. c) Manhattan plot displaying -log_10_ of the p-values. d) Q-Q plot showing that many p-values do not follow expected uniform distribution confirming outliers.

**
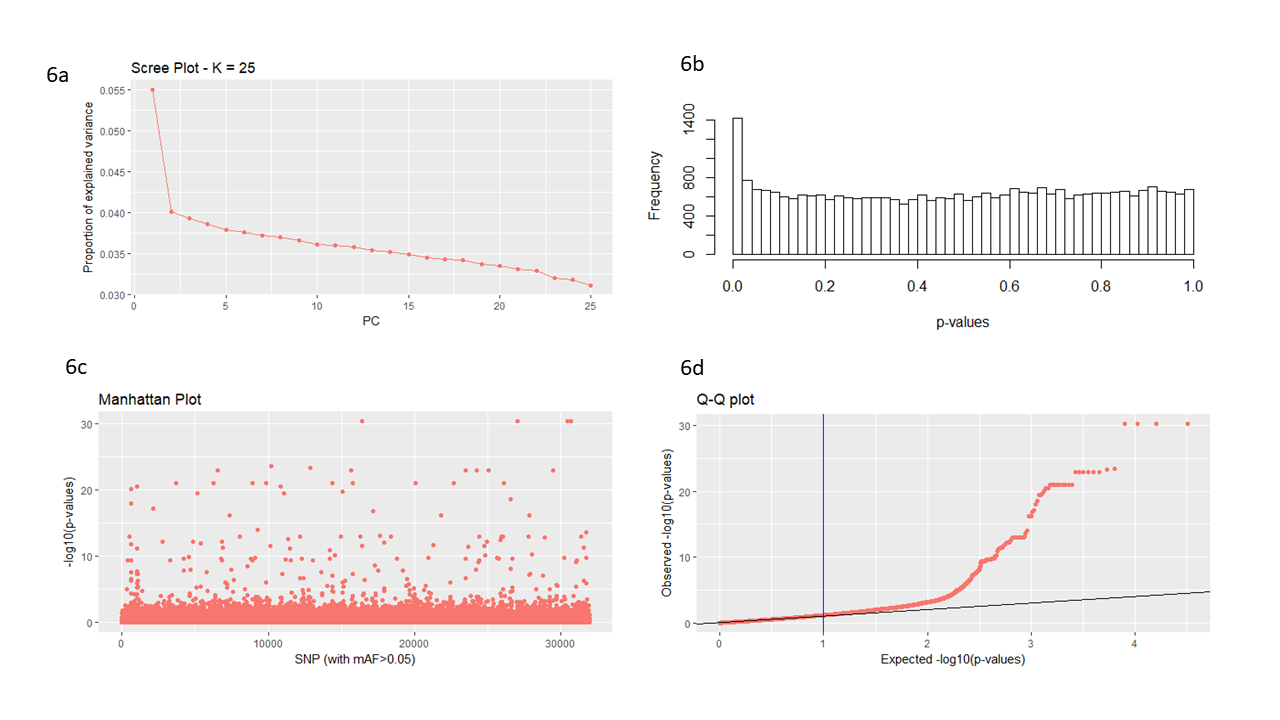
**

**Supplementary Figures 5a-d). Figures related to selection test for the Scandinavian genomic dataset (two populations) with PCadapt.** a) Scree plot: K=2. See explanation for figures b-d from Supp. Fig. 5.

**
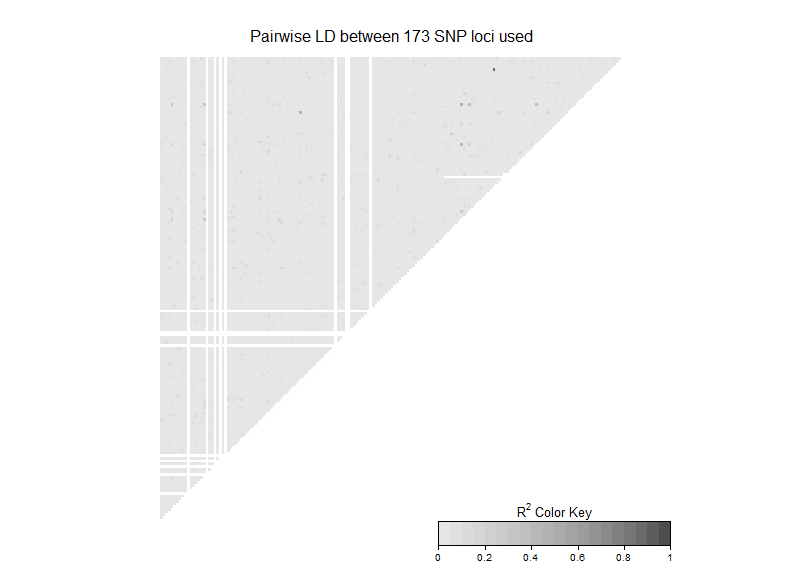
**

**Supplemenetary Figure 7.** Heatmap of linkage between the used 173 SNP loci**.** White lines are loci with lot of missing information (≥20% in one or both populations).


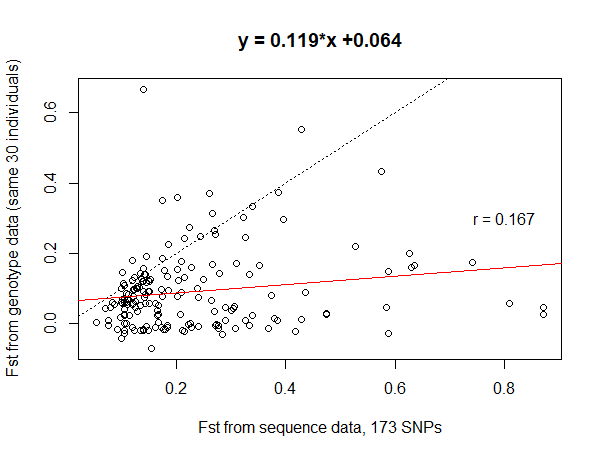


**Supplementary Figure 8a.** Expected *F_st_* between Scandinavian goldsinny wrasse populations based on 173 SNPs derived from sequencing data from 30 individuals (15 from each population) vs observed *F_ST_* based on genotyping of the same individuals. Regression equation is given above the figure. Red line shows the realized regression line, dotted line indicates perfect linear correlation. *R^2^*=0.028, *p*=0.029.


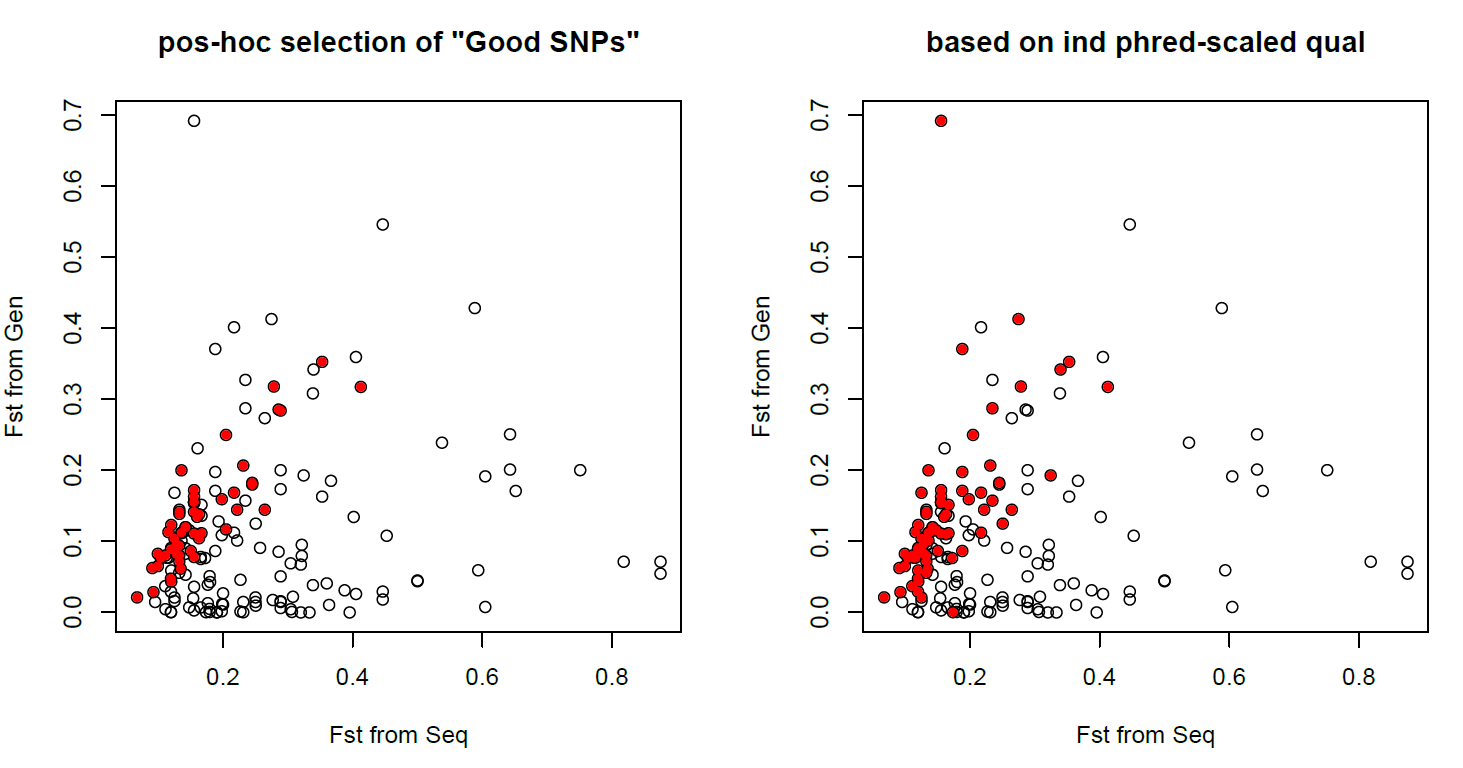


**Supplementary Figure 8b. S**election of SNPs based on additional filtering steps (see text for details). SNPs passing filtering steps are shown in red. Figure on left indicates SNPs with >90% genotype match between methods (*N*=51), whereas on right SNPs (*N*=74) with the best mean phred-scaled quality are shown. For the linear correlation test of these markers, see Supplementary Figure 8c below.


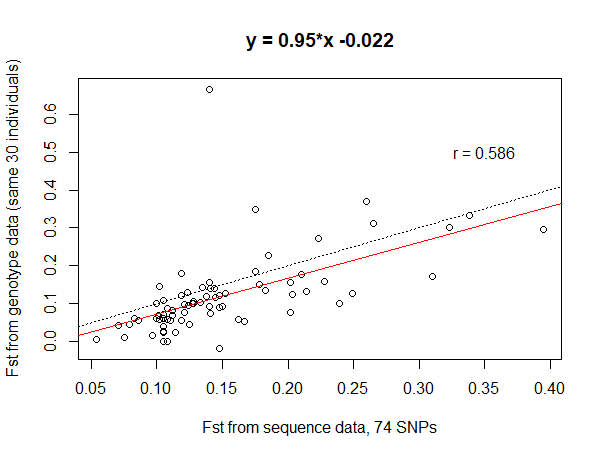


**Supplementary Figure 8c.** Expected *F_st_* between Scandinavian goldsinny wrasse populations based on 74 SNPs derived from sequencing data from 30 individuals (15 from each population) vs observed *F_ST_* based on genotyping of the same individuals. Regression equation is given above the figure. Red line shows the realized regression line, dotted line indicates perfect linear correlation*. R^2^*=0.344, *p*<0.001.


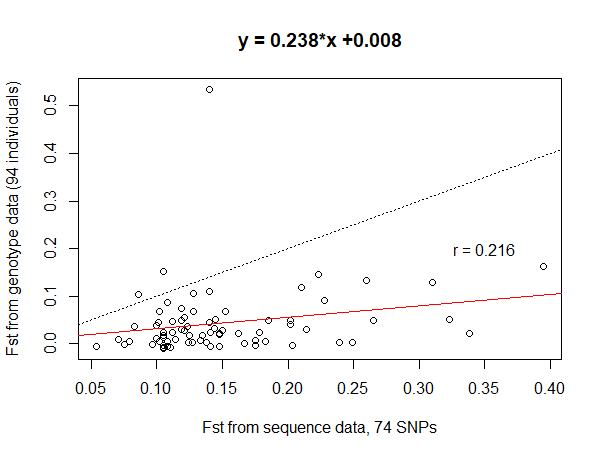


**Supplementary Figure 8d.** Expected *F_st_* between Scandinavian goldsinny wrasse populations based on 74 SNPs derived from sequencing data from 30 individuals (15 from each population) vs observed *F_ST_* based on genotyping of 94 individuals (47 from each population). Regression equation is given above the figure. Red line shows the realized regression line, dotted line indicates perfect linear correlation*. R^2^*=0.047, *p*=0.0642.


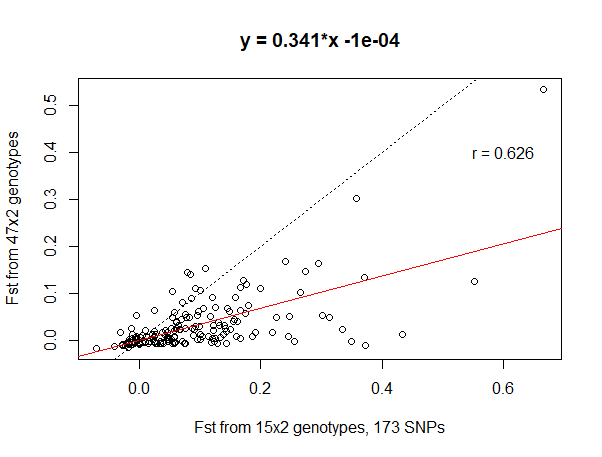


**Supplementary Figure 8e.** Observed *F_st_* between Scandinavian goldsinny wrasse populations based on genotyping of 30 (15x2) and 94 (47x2) individuals with 173 SNPs. Regression equation is given above the figure. Red line shows the realized regression line, dotted line indicates perfect linear correlation*. R^2^*=0.391, *p*<0.0001.


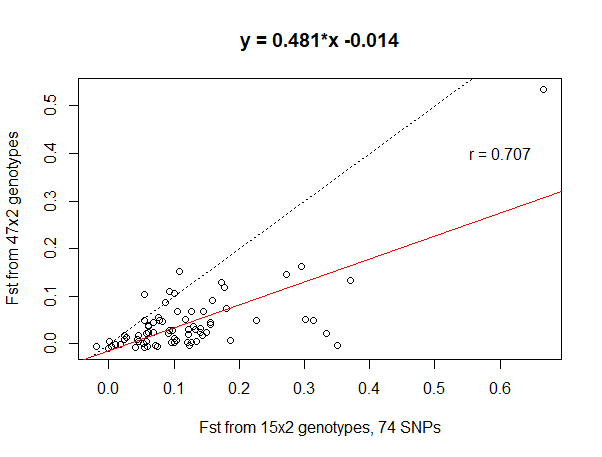


**Supplementary Figure 8f.** Observed *F_st_* between Scandinavian goldsinny wrasse populations based on genotyping of 30 (15x2) and 94 (47x2) individuals with 74 SNPs. Regression equation is given above the figure. Red line shows the realized regression line, dotted line indicates perfect linear correlation*. R^2^*=0.500, *p*<0.0001.

**
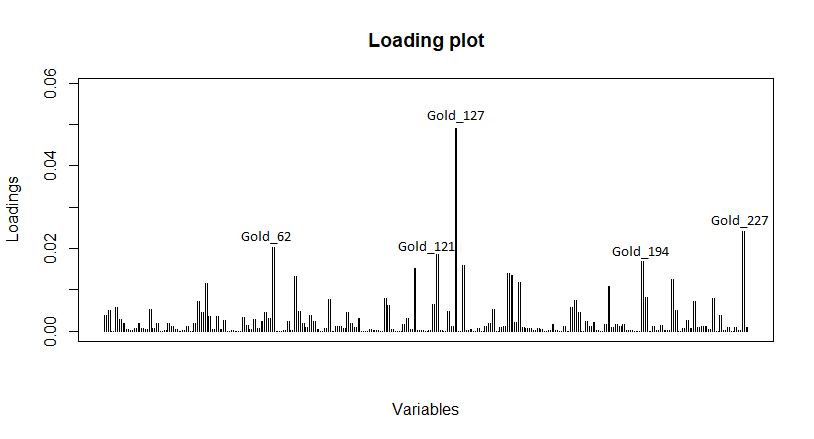
**

**Supplementary Figure 9. Loading plot showing individual locus contribution of the genetic divergence between the Scandinavian goldsinny wrasse populations.** Five most affecting loci are shown. GenBank® nucleotide searches for these SNP sequences did not give any hits.

**
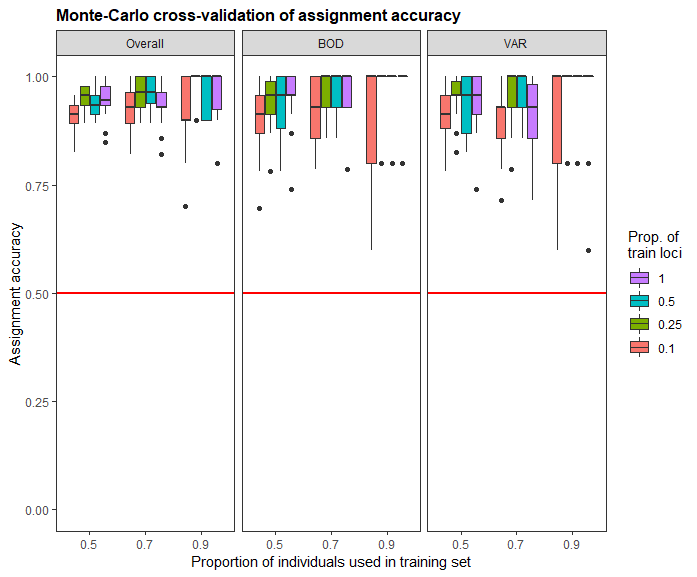
**

**Supplementary Figure 10. Assignment accuracy with different proportions of the 173 SNP loci based on Monte Carlo resampling procedure.** Red horizontal line show null assignment power for two populations.
